# Enhancing Communication about Paediatric Medicines: Lessons from a Qualitative Study of Parents' Experiences of Their Child's Suspected Adverse Drug Reaction

**DOI:** 10.1371/journal.pone.0046022

**Published:** 2012-10-10

**Authors:** Janine Arnott, Hannah Hesselgreaves, Anthony J. Nunn, Matthew Peak, Munir Pirmohamed, Rosalind L. Smyth, Mark A. Turner, Bridget Young

**Affiliations:** 1 Institute of Psychology, Health and Society, University of Liverpool, Liverpool, United Kingdom; 2 Alder Hey Children's National Health Service Foundation Trust, Liverpool, United Kingdom; 3 Institute of Translational Medicine, University of Liverpool, Liverpool, United Kingdom; 4 Institute of Translational Medicine, Liverpool Women's National Health Service Foundation Trust and University of Liverpool, Liverpool, United Kingdom; University of Edinburgh, United Kingdom

## Abstract

**Background:**

There is little research on parents' experiences of suspected adverse drug reactions in their children and hence little evidence to guide clinicians when communicating with families about problems associated with medicines.

**Objective:**

To identify any unmet information and communication needs described by parents whose child had a suspected adverse drug reaction.

**Methods:**

Semi-structured qualitative interviews with parents of 44 children who had a suspected adverse drug reaction identified on hospital admission, during in-patient treatment or reported by parents using the Yellow Card Scheme (the UK system for collecting spontaneous reports of adverse drug reactions). Interviews were conducted face-to-face or by telephone; most interviews were audiorecorded and transcribed. Analysis was informed by the principles of the constant comparative method.

**Results:**

Many parents described being dissatisfied with how clinicians communicated about adverse drug reactions and unclear about the implications for their child's future use of medicines. A few parents felt that clinicians had abandoned their child and reported refusing the use of further medicines because they feared a repeated adverse drug reaction. The accounts of parents of children with cancer were different. They emphasised their confidence in clinicians' management of adverse drug reactions and described how clinicians prospectively explained the risks associated with medicines. Parents linked symptoms to medicines in ways that resembled the established reasoning that clinicians use to evaluate the possibility that a medicine has caused an adverse drug reaction.

**Conclusion:**

Clinicians' communication about adverse drug reactions was poor from the perspective of parents, indicating that improvements are needed. The accounts of parents of children with cancer indicate that prospective explanation about adverse drug reactions at the time of prescription can be effective. Convergence between parents and clinicians in their reasoning for linking children's symptoms to medicines could be a starting point for improved communication.

## Introduction

Like all patients, children are at risk of adverse drug reactions (ADRs). We define an ADR as a “*harmful or unpleasant reaction, resulting from an intervention related to the use of a medicinal product, which predicts hazard from future administration and warrants prevention or specific treatment, or alteration of the dose regimen, or withdrawal of the product* [1].” Our use of the term ADR in this paper also follows that of the World Health Organisation and UK Medicines and Healthcare Products Regulatory Agency (MHRA), in that our focus was on reactions which occurred at normal therapeutic doses [2], [3] and were suspected by clinicians or parents to be related to the action of a medicine. ADRs may be distinguished from adverse medical events, which are untoward occurrences that may be present during medicinal treatment but which are not necessarily related to the action of the medicine, and may include errors in diagnosis, treatment and management that result in harm to the patient [1].

Evidence suggests that patients are generally poorly informed about medicines and the systems to ensure drug safety [4], [5]. The literature on communicating about medicines indicates the advantages of involving patients in open discussions about the potential benefits and risks of medicines at the time these are prescribed [6]–[11], the importance of such discussions for informed consent and decision making [6], [8], [10]–[13], and the value of supplementary verbal and written information tailored towards the needs of patients [7]–[9], [14]. The literature also highlights the complexities involved in communicating with patients about the uncertainties associated with medicines [8], [11]–[13], [15] and describes the relative merits of different methods of presenting information on risk and uncertainty, such as the use of numerical probabilities and frequencies [16], [17], and who should communicate information about risks of medicines [18].

This growing body of literature provides valuable general guidance for practitioners in communicating openly with patients about medicines. Providing patients with information about medicines is important. However, there are also concerns that informing patients about ADRs prospectively can induce expectations that leave patients more susceptible to experiencing ADRs and less likely to adhere to treatment [19]. Furthermore, untoward events during an illness may also be misattributed to medicines by patients and others [20].

The promotion of treatment adherence has driven much research and theory on beliefs and communication about medicines [21]. Evidence and theory suggest that patient adherence to a medicine is influenced by their beliefs [22]–[24]. The necessity-concerns framework [25] proposes that uptake and adherence to particular medicines is influenced by how patients evaluate the need for that medicine relative to their concerns about ADRs. At a more general level, patients' evaluations are also thought to be influenced by cultural beliefs, such as beliefs that medicines are harmful or overused [26]. More recently, attention has focussed on the concept of perceived sensitivity to the effects of medicines, that is, how responsive or susceptible patients perceive themselves to be to the effects of medicines, and how these perceptions of sensitivity influence patients' concerns about potential ADRs, reporting of ADRs and medication adherence [19], [27].

While efforts to optimise treatment adherence have driven much of the work on beliefs and communication about medicines [21], and optimising adherence is an important objective, less attention has focussed on enhancing communication about medicines as an important goal in its own right. In particular, little attention has been paid to the lessons that can be learnt from patients' accounts of experiencing ADRs and how these lessons can contribute to enhancing communicating about medicines and adherence. Moreover, little is currently known about the particular experiences and needs of child patients and their parents following the occurrence of a suspected ADR. Research has examined parents' perceptions of risks associated with child vaccines, but this has focussed on ways to promote adherence to vaccine schedules, rather than on parents' experiences of care in the aftermath of a suspected ADR [28]–[32]. Bellaire *et al* report that children with multiple ADRs following antibiotic treatment may experience lower health-related quality of life (HRQL) as perceived by parents, than healthy children or those with only one ADR [33]. However, interpretation of these findings is difficult as children with multiple ADRs in this study had significantly more co-morbidity than the other groups, so non-ADR related factors cannot be excluded as an explanation for the lower HRQL among the multiple ADR group [33]. Bellaire *et al* also describe anecdotal comments from participating parents suggesting that children's ADRs are a significant source of concern for parents [33].

Evidence that members of the public are particularly concerned about children's medicines comes from a study comparing lay people's responses to hypothetical scenarios involving medicines for child or adult patients. Respondents perceived the risks of ADRs to be more severe and reported that they would be less likely to take (or give) a medicine when the recipient was a child rather than an adult [9]. Adult patients receiving treatment for acute conditions and admitted to hospital with an ADR were frustrated and frightened by the experience [34], yet the situation of parents of child patients is likely to be further complicated by the frequent prescribing of unlicensed and off-label drugs in paediatrics [35]–[37] and by parents' distinctive role in caring for their children [38], [39].

Clinicians are encouraged to consider ADRs during the evaluation of every patient they see. There are established ways to assess the possibility that a drug has caused a harmful or unpleasant event. Although these causality assessments differ in their details, they contain some common elements. For example, the widely used Naranjo scale [40] includes questions such as “Did the adverse event appear after the suspected drug was administered?”, “Did the adverse reaction reappear after the drug was readministered?” and “Are there alternative causes (other than the drug) that could on their own have caused the reaction?”. These questions should be part of the clinical reasoning that clinicians use when they encounter a child with a potential ADR. The questions may be implicit or explicit but they are available to the clinician during their assessment. In principle, these questions are also available to the clinician when structuring their communication with children and their parents. It is unclear whether clinicians share their reasoning with parents and children.

In summary, there is a need to consider communication about ADRs as an important objective in its own right and in the context of optimising adherence. There are particular concerns that seem to surround children's medicines. Clinicians have access to structured approaches to dealing with suspected ADRs. Nevertheless, little is known about the experiences of parents when their child has had a suspected ADR. This means that clinicians have little evidence to guide them when communicating with families in the aftermath of a suspected ADR.

To inform the management of communication about ADRs in children we investigated parents' experiences of suspected ADRs in their child. Our focus was to identify any unmet psychological, information and communication needs described by parents. Given the absence of previous research in this area, we designed our qualitative study, ADRIC-QUAL, to explore all aspects of parents' experiences and views, from their accounts of communication at the point at which medicines were prescribed, to their views about the implications of ADRs for their child's future health [41].

## Methods

### Ethics statement

A UK National Health Service research ethics committee approved the study (Northwest 3 Research Ethics Committee 08/H1002/7). All participants gave written informed consent.

### Sampling, setting and recruitment

As recommended in qualitative research when there has been little previous research on a topic, we sampled for maximum variation [42], [43]. We used two sampling routes to ensure diversity in terms of ADR type and severity, and clinical speciality. The first route comprised two cohort studies. These were part of the Adverse Drug Reactions in Children (ADRIC) programme conducted at a regional paediatric hospital in the UK [44]. In particular, two studies within the ADRIC Programme (ADRIC1 and ADRIC2) investigated the prevalence of suspected ADRs among all patients aged less than 17 years; ADRIC1 focussed on patients with an unplanned hospital admission, while ADRIC2 focussed on patients admitted for 48 hours or more. ‘ADRIC families’ were eligible for ADRIC-QUAL if they could be approached about the study before discharge. They were not eligible if the family were experiencing pronounced distress or there were child protection concerns. Clinicians, hospital managers and the Ethics Committee concurred that approaching families in the latter two groups could complicate an already sensitive clinical situation and could not be justified for this research project. Treating clinicians initially introduced ADRIC-QUAL and interviewers subsequently provided interested families with more detailed information and contacted families post-discharge to arrange the interview.

We suspected ADRIC patients' ADRs were probably at the more severe end of the spectrum so we used a second sampling route, the Yellow Card Scheme [3], in order to maximise variability [42], [43]. Using the Yellow Card Scheme also enabled us to access a sub-sample of parents without the potential influence of clinician gate-keeping (in route 1 there was a possibility that treating clinicians may have declined to invite eligible ADRIC parents to participate, for example, due to reluctance to discuss ADRs with parents or because of perceived difficulties in the parent-clinician relationship). The Yellow Card Scheme is a national drug surveillance system which allows patients and families (as well as clinicians) to spontaneously report suspected ADRs directly to the UK competent authority for medicines approval and monitoring, the MHRA. Initially, the MHRA sent study invitation letters to all parents who had submitted a Yellow Card (YC) on behalf of a child under 17 years. However, in the first six months most parents were reporting suspected ADRs linked to vaccines, so thereafter only parents submitting YCs about non-vaccine related ADRs were sent letters. The letters outlined the study and invited parents to return a reply slip to the ADRIC-QUAL team if they wished to participate. The ADRIC-QUAL interviewer then telephoned parents to further explain the study and arrange an interview.

Sampling ran in parallel with data analysis, and was discontinued when saturation on the main analytical categories was reached [45].

### Interviews

Interviewers explained their independence from clinical teams and the MHRA before all interviews. JA and HH conducted face to face interview with ADRIC parents. YC parents resided across the UK, so JA, HH and ES conducted telephone interviews with them. Interviews were semi-structured and informed by a topic guide that contained prompts about families' experiences of: signs and symptoms in their child and how they linked these to a medicine; awareness of suspected ADRs; written and verbal communication with clinicians and views about the implications of ADRs for children (see Table S1 ‘Parents’ generic interview guide'). Interviewers tailored their questions to ensure interviews were conversational, and previously unanticipated topics were explored as interviewing and analysis progressed.

All audio-recorded interviews were transcribed. Transcripts included indicators of hesitation, repetition, dysfluency and sub-verbal vocalisations and were checked by the interviewer who removed all identifying details before analysis. Field notes were also recorded detailing the interview context, such as the setting of the interview, and observations and reflections on the interview process, including participants' interaction, demeanour and significant non-verbal behaviour.

### Analysis

Researchers point to the value of flexibility in inductive research and the importance of ensuring that the aims of a particular study guide the methods, rather than the reverse [46]–[49]. In this study, our overarching aim was to inform practice. While our analysis drew on methods associated with grounded theory such as constant comparison [41], [45], [50]–[54], we selected and applied these methods to fit with our focus on informing practice and the criterion of catalytic validity, whereby the findings should not merely describe, but have the potential to inform research and practice [55], [56]. Our orientation to the analysis was broadly interpretive that is, while our focus was parents' experiences, we did not simply take their accounts at face value, rather we considered how parents constructed their experiences and what was latent or deemphasised in their accounts, as well as the manifest content [57]–[60].

JA led the analysis and development of the coding framework in a process that had both inductive and deductive aspects. She read transcripts several times to develop analytic categories regarding the content and meaning of particular transcript sections. To avoid a fragmented or decontextualised analysis [61] and ensure that the analytical categories and developing analysis were consistent with participants' overall stances in their interviews, JA referred to the interviews as a whole [59]. This is important as the meaning of a transcript section might only become apparent by considering a participant's narrative as it develops over an entire interview. Similarly, JA also referred to the field notes during the analysis to prompt her recollection of contextual and process aspects of the interview and use these to help interpret the transcript sections. For example, field note reflections on a participant's interaction and the emotional ‘tone’ of the interview assisted in interpreting sections of transcript that might otherwise be ambiguous or misinterpreted. BY and MT supported the analysis by reading a sample of the transcripts and by ‘testing’ and developing the analysis through periodic discussion with JA. All three analysts compared within and between transcripts, and iterated between developing analytical categories and new data [41], [51]–[54], [61], [62], [63]. We did not use a qualitative data analysis software to assist the analysis, as we found the functions in Microsoft Word adequate [64]. However, we employed a number of methods that are recommended to help ensure rigour in the analysis of qualitative data. We used respondent validation, whereby we discussed the emerging analysis with later participants [41], [62]. We also attended to exceptional or ‘deviant’ cases, that is, cases that were untypical either because of the patient demographic or disease profile or because of the families' experiences, and examined how differences between these and more typical cases could inform the data analysis [51]–[54], [62], [63]. Finally, we scrutinized the quality of the developing analysis according to its coherence and, as noted above, its potential to influence practice, a process that was assisted by discussion among all authors [62], [65]. This multi-disciplinary investigator triangulation aimed to ensure the quality and clinical relevance of the analysis [66], [67]. We present brief data extracts in the main text of the results to illustrate key findings and supplement these with data extracts in boxes to evidence our interpretations of parents' accounts. Extracts are italicised and coded “A” (ADRIC parents) or “YC” (Yellow Card parents). Omitted speech is indicated by […]; explanatory text by [text].

## Results

### Participants

We conducted audiorecorded interviews with 45 parents of 44 children (41 mothers, four fathers). Of the 27 ADRIC families, 10 were recruited via ADRIC1 and 17 via ADRIC2. A total of 21 ADRIC2 families were approached to participate: three declined because of the child's repeated hospital admissions, and one further family was interviewed but declined to be audiorecorded. We were unable to record the number of ADRIC1 families who were approached to participate in ADRIC-QUAL by treating clinicians. Fifty-four YC families were sent MHRA invitation letters. Details of non-responders are not available, but of 21 who replied, we had audiorecorded interviews for 17. Of the four remaining YC parents, one could not be contacted and interviews with three were not audiorecorded due to equipment failure. Therefore, a total of four interviews had not been audiorecorded. Our only record of these was the fieldnotes made by the researchers after the interviews had taken place. Because these fieldnotes were considerably less detailed than the transcribed recordings and did not contain verbatim speech, we did not consider the fieldnotes to be equivalent to the transcribed recordings or treat them as such in the analysis. However, our review of the fieldnotes for the non-audiorecorded interviews indicated that they were consistent with the findings from the transcribed interviews.

Interviews lasted approximately 60 minutes (ranging from 20 to 100 minutes) and were conducted between 2–56 weeks after the suspected ADR. Three ADRIC parents were interviewed in private rooms in the hospital; the remainder were interviewed in their homes. All YC parents were in their homes during the telephone interviews. The Index of Multiple Deprivation scores of Yellow Card participants indicated less deprivation among this group than the ADRIC participants. Table 1 shows demographic characteristics for participants. To indicate the clinical context of each child, Table 1 also shows (as reported by parents) the type of medicines children had taken and the body system affected by the ADR. Where relevant, hospitalisation details and the body system affected by any underlying conditions that children had are also shown. Of the 26 ADRIC children whose suspected ADRs were classified using the Liverpool Causality Assessment Tool as part of the ADRIC 1 and 2 studies [68], three (11%) were deemed unlikely to have had an ADR, four (15%) were possible ADRs, 11 (41%) were probable and eight (30%) were definite. Data on ADR classifications are not available for one (4%) remaining ADRIC child or for the YC children.

**Table 1:** Children's demographic characteristics, medicine type and ADR information.

ID	Age^1^	Gender	Ranked IMD scores^2^	Type of drug associated with suspected ADR	Body system affected by suspected ADR	Severity score^3^	Whether suspected ADR contributed to hospitalisation/prolonged inpatient stay	Underlying condition by body system
A1	3–5	Female	403	Antibiotics	Skin and mucous membranes	3	Yes (contributed towards admission)	Respiratory
A2	12+	Male	10787	NSAID	Musculoskeletal	3	Yes (reason for admission)	Musculoskeletal
A3	3–5	Male	306	Corticosteroids, Cytotoxics	Haematological	3	Yes (reason for admission)	Haematological
A4	12+	Female	2482	Cytotoxics	Gastrointestinal	3	Yes (reason for admission)	Haematological
A5	0–2	Female	12821	Antibiotics	Skin and mucous membranes	3	Yes (reason for admission)	Respiratory
A6	0–2	Male	1574	Cytotoxics	Haematological	3	Yes (reason for admission)	Haematological
A7	0–2	Male	15485	Corticosteroids, Cytotoxics	Haematological, immune system	3	Yes (reason for admission)	Haematological
A8	3–5	Female	383	Vaccines	Skin and mucous membrane	3	Yes (reason for admission)	None
A9	6–11	Male	6091	Corticosteroids	Immune	3	Yes (reason for admission)	Musculoskeletal
A10	0–2	Female	12223	Vaccines	Immune Infection	Missing	Missing	Gastrointestinal
A11	6–11	Female	16778	Antibiotics	Skin and mucous membranes	1	No (inpatient stay not prolonged)	Musculoskeletal, nervous, respiratory, gastrointestinal, skeletal
A12	3–5	Female	271	Antiepileptic	Hepatic	3	No (inpatient stay not prolonged)	Musculoskeletal, nervous, gastrointestinal
A13	0–2	Male	N/A	Antibiotics	Skin and mucous membranes	3	No (inpatient stay not prolonged)	Musculoskeletal, gastrointestinal, nervous
A14	6–11	Male	19865	Opioid analgesia	Nervous	3	No (inpatient stay not prolonged)	Gastrointestinal
A15	12+	Female	24299	Opioid analgesia+other post-operative analgesia	Nervous	3	No (inpatient stay not prolonged)	Musculoskeletal
A16	0–2	Female	24447	Opioid analgesia	Skin and mucous membranes	3	No (inpatient stay not prolonged)	Gastrointestinal
A17	12+	Male	108	Opioid analgesia	Gastrointestinal	1	No (inpatient stay not prolonged)	Gastrointestinal
A18	12+	Male	N/A	Antibiotics	Manifestation was flushing of skin but underlying cause was immune	3	No (inpatient stay not prolonged)	Musculoskeletal, skin and mucous membranes, renal, gastrointestinal, metabolic
A19	0–2	Male	18461	Antibiotics	Manifestation was flushing of skin but underlying cause was immune	3	No (inpatient stay not prolonged)	Gastrointestinal
A20	6–11	Female	14971	Drugs used in status epilepticus	Nervous	1	No (inpatient stay not prolonged)	Gastrointestinal
A21	12+	Male	19823	Opioid analgesia	Gastrointestinal	3	No (inpatient stay not prolonged)	Musculoskeletal
A22	6–11	Male	29022	Opioid analgesia	Respiratory	1	No (inpatient stay not prolonged)	Musculoskeletal, nervous
A23	3–5	Male	5171	Opioid analgesia	Gastrointestinal	3	No (inpatient stay not prolonged)	Cardiovascular
A24	12+	Male	N/A	Corticosteroid	Cardiovascular	5	No (inpatient stay not prolonged)	Haematological
A25	6–11	Male	26028	Opioid analgesia	Nervous	3	No (inpatient stay not prolonged)	Musculoskeletal, nervous
A26	12+	Female	11667	Drugs affecting the cardiovascular system	Nervous	3	No (inpatient stay not prolonged)	Musculoskeletal, cardiovascular
A27	6–11	Female	24071	Antibiotics; Non-opioid analgesia	Skin and mucous membranes	3	No (inpatient stay not prolonged)	Respiratory
YC1	12+	Male	32210	Immunological products and vaccines	Endocrine	N/A	N/A	None
YC2	12+	Male	17251	Drugs used for attention deficit disorder	Neurological	N/A	N/A	Mental health
YC3	12+	Female	20387	Immunological products and vaccines	Haematological	N/A	N/A	None
YC4	12+	Male	31691	Non-opioid analgesia	Renal	N/A	N/A	None
YC5	12+	Female	20737	Immunological products and vaccines	Neurological, Musculoskeletal, Gastrointestinal, Skin and mucous membranes, mental health	N/A	N/A	None
YC6	12+	Female	31439	Immunological products and vaccines	Neurological, Immune, Musculoskeletal	N/A	N/A	None
YC7	6–11	Female	N/A	Respiratory	Mental health	N/A	N/A	Respiratory
YC8	12+	Female	29831	Immunological products and vaccines	Musculoskeletal, Neurological	N/A	N/A	None
YC9	6–11	Male	22922	Immunological products and vaccines	Gastrointestinal	N/A	N/A	None
YC10	12+	Female	30656	Immunological products and vaccines	Neurological, Musculoskeletal, Immune	N/A	N/A	None
YC11	0–2	Male	31508	Immunological products and vaccines	Haematological	N/A	N/A	None
YC12	12+	Female	30775	Immunological products and vaccines	Immune, neurological	N/A	N/A	None
YC13	2–6	Male	9436	Respiratory	Behavioural changes	N/A	N/A	Respiratory
YC14	2–6	Male	31612	Respiratory	Behavioural changes	N/A	N/A	Respiratory
YC15	6–11	Male	29750	Drugs used for attention deficit disorder	Neurological	N/A	N/A	Mental Health
YC16	12+	Male	25366	Insulin	Behaviour changes, gastrointestinal	N/A	N/A	Endocrine
YC17	6–11	Female	15380	Antibiotic	Skin and mucous membranes	N/A	N/A	None

1 Age reported in year groups: 0–2; 3–5; 6–11; 12 years and over.

2 Calculated using Lower Super Output Area (LSOA) 2007 ranked score data, whereby lower scores indicate greater deprivation (data for families outside England are not reported due to incompatibility between IMD scoring systems within UK).

3 Severity scores were assessed using the Hartwig scale [84] where 1 = No change in treatment with suspected drug; 2 = Drug dosing or frequency changed, without antidote or treatment for exhibited symptoms; 3 = Required treatment, or drug administration discontinued; 4 = Resulted in patient transfer to higher level of care; 5 = Caused permanent harm to patient or significant haemodynamic instability; 6 = Directly or indirectly resulted in patient death.

The format in which we present our findings broadly reflects the main categories that we developed during the course of the analysis. We first describe parents' accounts of communication about medicines at the time of prescription and how they first became aware that their child's symptoms might be linked to a medicine. We then describe parents' accounts of seeking help for their child's symptoms and their experiences of communication with clinicians when an ADR came to be suspected, the implications this communication had for parents' sense of involvement in their child's care, their perceptions regarding how their child's future care needs would be addressed, and how the experience of a suspected ADR influenced parents' views about children's medicines. Finally, we present parents' suggestions regarding how communication could be enhanced to better address their needs and those of their child.

### Little explanation of the risks of medicines at the time they were prescribed

Most parents indicated that clinicians tended not to explain the risks of medicines when the medicines were prescribed: *“No side-effects were made known to me”* (YC5). Parents explained how clinicians focussed on other issues, such as explaining their child's condition and the importance of medicines or surgery in treating the condition: *“They* [the surgeons] *don't discuss the drugs; they discuss the surgery itself”* (A23). If the risks of medicines were discussed, it was often at a time when parents struggled to absorb information, such as shortly before a child was due to be anaesthetised: *“On the day your child is being operated on or when the anaesthetist comes up you are not thinking of anything other than […] what's going to happen in the operation”* (A16).

Parents also reported difficulties with written information about medicines and potential ADRs. They either did not receive these documents: *“No information leaflet was given to me”* (YC5); *“You only get the bottle from the doctor don't you?”* (A1) or found them hard to engage with because the documents were too lengthy or did not seem relevant to their own child: *“I did a carefree glance* [at the patient information leaflet] *and chucked it”* (YC13). A key exception to these accounts of poor communication was the parents of children with cancer , who described how clinicians provided comprehensive information about the types of reactions that medicines could cause and emphasised how clinicians carefully timed and paced their explanations so that parents could absorb the information: *“They explained things in little bits so it sinks in […] they did say he would become neutropenic”* (A6). Parents of children with cancer also commented on how they were regularly asked about any medicine related difficulties their children were experiencing.

### How parents become aware that their child may be experiencing an ADR

Parents usually described an initial period in which they began to suspect something was wrong with their child based on a wide collection of physical symptoms and changes in behaviour that were ‘out of the ordinary’. With the exception of parents of children whose suspected ADR had first been identified by clinicians or whose children had cancer, parents initially tended to attribute symptoms to trivial causes such as minor illness, injury, or changes in their child's life or environment. It was only when symptoms worsened that parents became concerned: “*His colour dropped and his breathing went a bit funny and he started to panic, that worried me*” (A25) and they started to consider possible links to medicines.

Parents reported how they linked their child's symptoms to a medicine when they noticed patterns in their child's symptoms, such as a temporal association between giving a medicine and the onset of symptoms: “*It just seems strange to me that she had it* [the medicine] *and then straight away like she got that temperature”* (A10); “*The bottle would be finished, and the next day she would come out in a rash”* (A1); *“It's too much of a coincidence […] she had a needle and then that happened. She had a vaccination and then she had that”* (A8). Some parents also noticed how their child's symptoms receded between doses: *“She wasn't sick all night and then the next two times she had the Penicillin she threw up near enough ten minutes, fifteen minutes later”* (A5); *“He was off it* [the medicine] *for a couple of days. And then on the Sunday we noticed that his behaviour wasn't as bad”* (YC14); *“I noticed a difference […] when she was having it* [the medicine] *and when she wasn't having it […] she started on it again and then we noticed the symptoms within a few days again of having it”* (YC7). The absence of an alternative explanation for their child's symptoms also influenced parents' attributions about their child's suspected ADR: *“She came out of hospital when she was born and she hasn't been anywhere. She hasn't* [had] *nothing- nothing like foreign in her body at all, until she went for that vaccination”* (A10); “[The medicine] *is the only thing she's had and she hadn't had a cold or been ill before it”* (YC10).

Outside oncology, parents also spoke about the information sources that they drew on when making attributions about their child's symptoms. This included their personal experience with medicines, media coverage of problems with medicines and the concerns of friends and family: “*A lot of friends decided against it* [the human papilloma virus vaccine] *because it was a new vaccine*” (YC3). Information on the Internet could be a source of considerable anxiety for parents: “*I was on the Internet looking at all kinds of, I was beside myself, comas and everything”* (A8).

### With few exceptions, parents were critical about ADR management and communication

In a context in which parents sometimes described being overwhelmed with fear about their child's symptoms: “*I was at my wits end. All sorts were going through my mind”* (A2), parents' communication needs could be extensive. However, parents' accounts indicated that clinicians' communication about a child's suspected ADR was often poorly matched to parents' needs: *“They don't communicate with you as well as they should do, by my opinion”* (A23). Parents described a lack of communication that might help them understand what was happening to their child while his or her symptoms were being assessed and how clinicians were managing their child's symptoms: *“No-one actually ever said why it* [the hallucination] *was happening, the nurses thought it was a bit funny, they all kept coming over to see him and laughing with him sort of thing”* (A14). They reported communication as being contradictory and poorly coordinated, with some clinicians attributing the child's symptoms to a medicine, while other clinicians attributed the same symptoms to different causes: “*The first man said it was herpes […] and then the nice doctor downstairs said, ‘No, this is a reaction to Penicillin’”* (A5).

Parents remarked that the way in which clinicians managed and communicated uncertainty surrounding an ADR's identification did little to reassure them: *“I was saying ‘well, when she goes home, can I give her paracetamol? Can she never have paracetamol or can she never have a drug that might affect her liver?’ And they were going ‘well […] it should be fine’ but no-one was saying ‘well you can, I'll write it down and you can have it’”* (A12). Parents also described how they found clinicians' communication was poorly timed and paced, with parents receiving detailed information at times when they were anxious (e.g. when a child was critically unwell or immediately prior to surgery) and it was hard to absorb information, and receiving little or no information at times when they were less anxious and better able to absorb information. Commenting on how he/she felt overwhelmed with information at the height of his child's illness but received little support when his/her daughter's condition improved, the parent quoted above also remarked: “*All of a sudden because her figures have gone down […]* [the doctors are] *out the way now”* (A12).

Some parents were intensely critical and one parent, who was frustrated during a visit to outpatients when clinicians could not explain what was happening to his/her child spoke of feeling that he/she was being lied to by clinicians: “*They were fobbing me off […] I felt like they were lying to us”* (A5). More commonly, parents emphasized how their concerns had been ignored or dismissed by clinicians: *“We mentioned that she's not taking the* [respiratory medicine] *anymore because of the symptoms and they didn't comment on it”* (YC7); *“Dismissive and wasn't taking me very seriously”* (A10).

While YC and ADRIC parents both voiced criticisms of clinicians' communication, YC parents were particularly emphatic in their criticisms, especially when they felt clinicians had dismissed the possibility that a child's symptoms could be related to a medicine with little exploration of parents' concerns or explanation of the reasons for ruling out an ADR: *“She* [GP] *literally said word for word ‘What would you like me to do?’ And I just felt that was really dismissive”* (YC14). The sense that their concerns had been ignored or dismissed by clinicians left parents feeling abandoned: *“I just, just felt like nobody cared, nobody was interested and they just wanted me to go away”* (YC5); *“I went away with all this inadequate information […] I thought we really don't know anything […] we were sent home without even knowing when we were going to speak to a professional”* (YC1).

A striking exception to these highly critical accounts came from the parents of children with cancer. As we outline in the next section, these parents were almost uniformly highly positive in their accounts of how clinicians communicated about ADRs.

### Parents of children with cancer were positive about ADR communication

Despite the life-threatening nature of the illness and the risks of cancer treatment, parents of children with cancer felt well supported by how clinicians communicated with them about medicines. There was a sense from the accounts of these parents that clinicians took ADRs seriously, were adept in communicating about them and had well-developed systems in place for the management of ADRs: “*It's quite scary when you first go home with this big bag of drugs […] they said […] you can ring any time, and I rang nearly every day”* (A7). Parents pointed to how clinicians discussed possible ADR symptoms and how to respond before an ADR happened, so that parents were clear about what to look out for and what action to take in the event of a suspected ADR. Consequently, parents felt that clinicians communicated about medicines and ADRs in a way that was ordered, timely and reassuring.

### Implications of poor communication about suspected ADRs

Other parents reflected on the implications of poor communication about medicines and suspected ADRs. Parents commented on how a lack of information about potential ADRs at the time of prescription had prevented them from being involved in decisions about their child's care: *“If somebody had have told me that it causes the wind […] and the constipation I probably would […] have been a bit more forceful and say ‘well shouldn't we give him this now?’”* (A23); *“If someone had explained maybe […] the reactions […] we might have thought a bit more about taking it wouldn't we?”* (A25). In one case, lack of information at the time of prescription had resulted in a parent continuing to give morphine to alleviate their child's agitation, only to subsequently discover that agitation could be a result of itchiness caused by morphine: “*As she kept getting more and more agitated we kept boosting it* [the morphine] *[…] and the more we pressed the booster[…] the itchier she got”* (A16).

Parents also spoke of fearing a repetition of the ADR: “*Will it happen again? […] could it happen to him, to the baby?”* (A8), and of their uncertainty about the implications of ADRs for their child's future health and use of medicines. A few parents remarked on how they blamed themselves for what had happened because they felt: “*Responsible for what goes into* [their child]. *I always think with these things ‘Oh, it's my fault’ […] Why did I let her go ahead with it?”* (YC10). This was a source of distress for some parents: “*I was devastated […] you think you're doing them good and then the next minute she's in hospital and she could be having operations […] I just felt like crying all the time”* (A8). Moreover, parents either assumed that responsibility for preventing a recurrence of the ADR was theirs alone: *“It's something that I […] have to ask to make sure he never gets given that again”* (A18), or they were unclear about whether clinicians would take responsibility for preventing a recurrence of the ADR: *“I don't know if it would be down to me to turn round and say something or whether they have actually put something in their notes”* (A14); *“If there was ever a situation where she didn't have nothing on her to say that she was allergic to morphine and something happened to her outside […] maybe I wasn't there […] I don't know what might happen”* (A11).

In the context of poor communication, the experience of a suspected ADR sometimes coloured parents' views about medicines, and some expressed reluctance to give certain medicines to their child in the future. One parent became convinced that her child's ADR was a reaction to morphine and that this meant her child could never have morphine again: *“She's due for this big operation and she can't have morphine*” (A11). However, clinical review of this particular case suggested that the suspected ADR was linked to an avoidable over dosage, and that rather than avoiding morphine altogether in future, it might be in the child's best interests to personalise the dose. Another parent explained how her son was “*reluctant*” (A25) to accept painkillers, despite being in pain because he feared a repetition of a reaction to the opioid analgesia that he had taken, while another parent refused to allow her child to have the final course of her vaccine: “*I will categorically say that […] I will definitely not let her have the third* [human papilloma virus] *vaccine”* (YC3).

### How parents thought communication about suspected ADRs should be handled

Reflecting parents' accounts of poor communication about ADRs and the resulting implications as described above, parents wanted clinicians to help them to understand what had happened to their child. One parent explained how the need to understand the event was intrinsic rather than motivated by ulterior considerations: *“*[It's] *not necessarily the case that everyone's going to jump and say, ‘Right, I'm going to sue the drug company’ and all of these sorts of things. I think parents genuinely, who are concerned about their child's health, want to know what it was”* (YC8). Another parent remarked on how regular contact with a clinician had been reassuring: *“The doctor was back every half an hour checking on him […] Just to reassure me that everything was alright, it was just a reaction from it and he will be fine”* (A18). Indeed, parents wanted discussions about what had happened to their child to be paced and timed in a way that would help them to absorb the information: “*You just don't think straight when you're there […] doctors have got to understand that […] and maybe spend a little more time to try and explain a little bit more than they do”* (A11).

As we also note above, parents wanted to understand what the suspected ADR meant for their child's future health care, and they wanted to know about what steps would be taken to help prevent their child suffering further ADRs to ensure s/he would receive appropriate medicines in the future. Without exception, parents accepted that a certain level of risk came with medicines and most appreciated that clinicians faced uncertainty in identifying ADRs: *“I think it was the antibiotics. The doctors think it is that but they can never say it is that, because there is a possibility that it's not that”* (A1); “*It's just something that, you know, just happens […] I'm sort of accepting about it”* (YC13); *“I think that ‘It's a possibility’ is fine, erm. As long as it's explained clearly”* (YC14). While parents sometimes thought clinicians were unwilling to discuss ADRs, none blamed clinicians for their child's problems or said they intended to formally complain, and only one expressed a slight “*loss of trust”* (YC8) in clinicians. However, a few parents explained that their trust in medicines had diminished. Alongside their wish for dialogue with clinicians about ADRs, several parents also wanted accessible and reliable written information about ADRs: *“A leaflet about morphine […] in layman's terms erm you know, these side effects are rare but do look out for these”* (A16);*“They should give you a little pamphlet or something to say […] look this is what she's got”* (A12); *“We get sheets from the pharmacy department […] it is something I can refer to and I would much rather that it was given via the treatment centres than looking on the Internet because the Internet can be a horrible place”* (A26)

## Discussion

Parents were generally disappointed with how clinicians communicated about suspected ADRs. The majority reported receiving little or no advance explanation about the problems that might be associated with medicines. When information was provided, it was in ways that parents found hard to absorb. As a result, parents were taken by surprise when their child experienced a suspected ADR. This turned into frustration and confusion when clinicians were unresponsive to parents' concerns and some parents felt dismissed or abandoned as a result. In the absence of explanation about what steps could be taken to prevent further ADRs, a few parents were reluctant to give their children medicines in the future. The key exception to these negative accounts was parents of children with cancer, who despite their intense fears about the illness and treatment, were generally highly satisfied with how clinicians communicated about ADRs.

Our findings are important because as well as being a source of avoidable parental distress, poor clinician-parent communication about suspected ADRs may challenge parents' confidence in medicines and contribute to negative perceptions and misunderstandings of medicines [69], [70]. This could lead to poor adherence in the future. We found considerable convergence among parents about the nature of helpful communication. Their suggestions were also similar to those reported elsewhere, particularly regarding the process of communication, such as the importance of the timing and pacing of information, as well as the need for clinicians to explicitly acknowledge what had happened to the child and help parents to understand events that they perceive to be significant, even if the event is not clinically significant from the perspective of clinicians [71]–[73]. The accounts of parents of children being treated for cancer indicate that, despite the complexities involved in prospectively explaining about ADRs whilst not raising undue alarm about medicines, communication about ADRs can be conducted in ways that parents find informative, understandable and reassuring. However, we cannot rule out the possibility that other factors besides communication, such as illness beliefs specific to parents of children with cancer, (whereby, for example, ADRs are tolerated as the ‘price’ of life-saving treatment), might contribute to the contrasts between the accounts of parents of children with cancer and the accounts of the other parents. Moreover, every child with cancer will experience ADRs as a result of their treatment and the clinicians caring for them will have experience of children who have had severe ADRs or died as a result of the treatment. Such experiences will undoubtedly influence the priority that clinicians caring for children with cancer give to ADRs and the way that they communicate about medicines. It would be unwise or unrealistic to suggest that clinicians in other specialties should provide parents with the intense support that parents of children with cancer receive. Equally, it would be nihilistic not to attempt to improve parents' experiences of communication about ADRs given the problems they currently report. While clinicians are likely to focus on prospectively briefing parents about ADRs in high-risk situations (e.g. oncology), there are opportunities to extend such briefings to other planned care settings where ADRs are predictable (e.g. anaesthetics).

Parents' wishes for a dialogue with clinicians during the evaluation of ADRs resonate with anecdotal parental comments reported by Bellaire *et al* indicating that parents prefer to see clinicians who appreciate the significance of children's ADRs [33]. The accounts of parents in our study also echo advice about adverse medical events. As we note in the introduction, adverse medical events differ from ADRs in that adverse medical events are not necessarily attributable to the action of the medicine, although the event may have happened during medicinal treatment. The guidance on adverse medical events emphasises the importance of openly acknowledging that a problem or error has occurred and timely and clear communication [8], [71], [72], [74]–[79]. While the adverse medical events literature offers some useful insights, we cannot automatically apply its lessons to guide clinicians on how to communicate about ADRs in children, particularly as the ADRs that we focussed on in this study were not the result of an error and much of this literature has focussed on adult patients rather than parents. Research is now needed to explore clinicians' views and experiences of suspected ADRs in children. If parallels are found between clinicians' accounts of communicating about ADRs and the literature on their experiences of communicating about AMEs, the methods used to improve communication about AMEs [14], [71], [80], [81] may offer strategies for improving communication about ADRs.

One important challenge facing clinicians who communicate about ADRs is the uncertainty involved in attributing symptoms to medicines. Findings from other clinical contexts where uncertainty is prominent [71], [72], [74]–[80], [82] may offer further insights on managing communication in a context of uncertainty. We found that parents' accounts of how they linked their child's symptoms to a medicine resembled the logic that clinicians use to assess the likelihood of ADRs. Similar to this logic and the reasoning that underpins tools for assessing ADRs [68], [83], parents noted temporal associations between a medicine's administration and the onset of symptoms, the receding of symptoms between doses, and the absence of alternative explanations for symptoms. Clinicians use similar questions to assess whether a reaction can be attributed to a drug. The accounts of parents in this study imply that clinicians did not share their reasoning with parents when assessing the likelihood of an ADR. Nevertheless, the resemblance in the logic that parents and clinicians use in attributing symptoms to medicines indicates some common ground between the two parties. This common ground could be a starting point for improving communication about ADRs. Alongside our other findings - parents accepted that all medicines come with risks, appreciated the uncertainty in attributing symptoms to medicines and did not blame clinicians for suspected ADRs - we think there is reason to be optimistic about the potential to improve clinician-parent communication about medicines. However, research is now needed to investigate clinicians' perspectives on communicating with parents about suspected ADRs to ensure recommendations are realistic and practicable. Among other issues, this could explore the factors that influence the timing and nature of clinicians' communications with parents about ADRs.

Our study had some limitations. First, we were unable to access data on eligible ADRIC families who were not approached or did not participate in interviews, and on YC parents who did not respond to the MHRA's letter. As a result, we can say little about how interviewed parents compare to those groups. Second, before approaching ADRIC parents we were required to consult with their clinical teams, which may have filtered out parents whose relationships with clinicians were strained. To address this we sampled YC parents, as we could access them without consulting with clinicians. However, many YC parents were health professionals themselves, or had contacts who were and their views on communication about ADRs may be distinctive. Nevertheless, the accounts of both ADRIC and YC parents triangulate in pointing to the difficulties parents experience in communication about ADRs. Finally, the interviews were conducted some time after the child's suspected ADR, which may have shaped their accounts in certain ways. However, understanding the meanings that parents take away from their experiences of ADRs is crucial in learning how to improve their experience of ADR management and it is these meanings that were the focus of our study.

### Conclusions

Poor communication about children's ADRs was a source of significant difficulty for parents. Our findings will help guide clinicians regarding what topics to cover in their discussions with families about medicines and ADRs. At the time of prescription, parents wanted to know the potential risks associated with medicines. In the aftermath of a suspected ADR, they wanted to understand what had happened to their child and in some cases this might include explicit acknowledgment that an ADR had possibly occurred. Parents also wanted know the potential future implications of the suspected ADR for their child. Parents linked their child's symptoms to medicines in ways that resembled the reasoning used clinically for identifying ADRs and clinicians could possibly use this common ground as a starting point for communicating with parents when an ADR is suspected. However, our study's most important contribution may lie in providing insight for clinicians into how valuable discussions of ADRs can be for parents.

## Supporting Information

Table S1 **Parents' generic interview guide.**
(DOCX)
