# Supplementary material for: Enhancing Communication about Paediatric Medicines: Lessons from a Qualitative Study of Parents' Experiences of Their Child's Suspected Adverse Drug Reaction
Source: PLoS One. 2012 Oct 10;7(10):e46022. doi: 10.1371/journal.pone.0046022 (PMC3468607; doi:10.1371/journal.pone.0046022)
Supplement: Table S1 — Parents' generic interview guide. (DOCX) [file pone.0046022.s001.docx]

**Table S1: Parents’ generic interview guide**

**Adapted for ADRIC and Yellow Card families**

**1. Introduction to project and child**

- 1. Recap of study aims and details.

1.2 Review of child’s current health.

*ADRIC parents*

- 1. Confirmation and review of child’s hospital admission and inpatient stay.

*Yellow Card parents*

- 1. Confirmation and review of the Yellow Card submitted by the parent.

**2. First signs/symptoms**

- 1. How parents first noticed their child was unwell.
  2. Parents early ideas about causation
  3. Who first noticed a problem?
  4. What happened on child’s admission/on the ward/at the GP surgery?
  5. Did parents link medicines and symptoms at this stage?

**3. Experiences on admission to hospital (ADRIC parents)**

3.1 Reviews of events in Accident and Emergency or on the hospital ward. Who was involved?

- 1. What were parents thinking at the time?

3.3 Could parents ask questions and who did parents talk to? Were their questions answered?

- 1. Anything else that parents can remember about the early signs of symptoms.

1. **Experiences in the community (Yellow Card parents)**

4.1 Did parents seek professional help for their child’s symptoms?

4.2 Ask parents to ‘walk through’ what happened

4.3 Who did parents talk to? How did they respond?

4.5 What were parents thinking at this stage?

4.6 Did anyone mention suspected problems with a medicine? Who was this?

4.7 What were parents told/advised about suspected problems with medicines?

4.8 What happened next?

**5. Experiences in hospital (ADRIC parents)**

5.1 Review details of child’s stay; length of stay; ward or unit. Did parents stay with their child?

- 1. Who was looking after their child? Did anyone discuss any suspected problems with medicines with the family? Who was this and what did they say?
  2. Could parents ask questions about suspected problems with medicines? Who did they talk to/what did they ask?
  3. What information did parents receive about suspected problems with medicines?

**6. Child’s Medicines**

*About all medicines*

- 1. Was the child taking any other medicine at the time?
  2. What was this and what was it for?

*About the medicine*

- 1. What happened when the medicine was prescribed? Was the parent there? Did the doctor/nurse/pharmacist explain what the medicine was for?
  2. Did parents receive any advice about the medicine, such as possible side effects? Was there any written information?
  3. Did the medicine seem to help?

*ADRIC parents*

6.6 Did any of the staff in hospital think there may be a problem with the medicine the child was taking? Who was this? What were parents told/advised?

- 1. What happened?

*Yellow Card parents*

6.7 Was the child’s medicine stopped? How was decision made?

6.8 Did a GP/nurse or pharmacist advise a parent to stop giving the medicine to their child?

*All parents*

- 1. Did parents think there was a problem with the medicine their child was taking? Who did parents discuss this with and what happened next?
  2. Were there any other problems, such as a child refusing a medicine?
  3. Has the child had any previous problems with medicines?
  4. What are parents’ general views about medicines/children’s medicines?

**7. Parents’ perceptions about the value and quality of other / written information about child’s health problems/medicines**

7.1 Did the parents receive any written information with their child’s medicine? Was it useful? Where else did parents look for information about medicines?

- 1. What /who has been the least/most useful source of information about the child’s medicine?
  2. Have parents changed their views about medicines?

7.5 Have parents had any follow up discussions with a doctor/nurse/pharmacists about the suspected problem their child had with the medicine.

**8. Communicating with children about medicines and the health problems**

- 1. Was the child involved in discussions about the medicines and suspected problems with a medicine
  2. Did the child have any questions or concerns?
  3. Who addressed these? What do parents think about involvement of child in discussions/decisions about medicine?

**9. Parents views on their child’s health now?**

- 1. What did parents find helpful? What could have been done differently?
  2. How do parents feel about medicines now? How would they respond if their child needed the same medicine again?
  3. Has anyone discussed with parents about whether their child definitely had a side effect? What do parents think?
  4. Do parents have any unanswered questions?
  5. How do parents think discussions with clinicians about ADRs should happen? When is the best time to discuss medicines?

9.6 What terms do parents use? (Adverse drug reaction, side effect, allergic reaction)

**10. General views and advice**

10.1 What advice would parents give to newly qualified doctors/nurses/pharmacists about talking to families about medicines?

10.2 What advice would parents give to other parents about children’s medicines?

**11. Provide an opportunity for parents to raise any other issues important to them.**
